# Essential Oil from the Leaves of *Chromolaena odorata*, and Sesquiterpene Caryophyllene Oxide Induce Sedative Activity in Mice

**DOI:** 10.3390/ph14070651

**Published:** 2021-07-06

**Authors:** Godfried Dougnon, Michiho Ito

**Affiliations:** Department of Pharmacognosy, Graduate School of Pharmaceutical Sciences, Kyoto University, 46-29 Yoshida-Shimoadachi-cho, Sakyo-ku, Kyoto 606-8501, Japan; dougnon.tchetonnougbo.26v@st.kyoto-u.ac.jp

**Keywords:** *Chromolaena odorata*, essential oil, caryophyllene oxide, *β*-caryophyllene, sedative effect, locomotor activity, inhalation

## Abstract

*Chromolaena odorata* (L.) R.M.King & H.Rob. essential oil (COEO) was investigated for its sedative activity in mice. The results showed that COEO significantly reduced mice locomotor activity and the most efficient concentrations were 0.04 and 0.00004 mg/cage (volume of the cage 61.2L). Analysis of chemical composition of the oil indicated that caryophyllene oxide (43.75%) was the major compound and bioactivity-guided fractionation of the oil was performed to isolate the compound responsible for activity. The data clearly identified sesquiterpene caryophyllene oxide as the compound inducing COEO sedative activity and it was effective in decreasing mice locomotor activity by 56% and 57% at 0.0004 and 0.04 mg/cage, respectively. In order to understand the action mechanisms, caryophyllene oxide was tested for its effects on the central nervous system (CNS) by using a caffeine pre-excited mice test and a pentobarbital sleeping-induced test in mice. The results showed that caryophyllene oxide is a potent CNS depressant. Nevertheless, it fails to potentiate the effects of pentobarbital on the GABAergic system, nor did flumazenil, a GABA_A_ receptor antagonist, reversed its effects. It was especially interesting to note that *β*-caryophyllene, the precursor of caryophyllene oxide, demonstrated a similar pattern of sedative activity, and the present work further extends actual knowledge on these naturally occurring sesquiterpenes. The findings in this study reveal the new activity of caryophyllene oxide as an innovative way to manage sleep and CNS-related disorders, and demonstrates a satisfactory effect of two interesting sesquiterpene compounds on the CNS.

## 1. Introduction

Essential oils have been used for their beneficial properties in human medicine, due to their abundance and diversity in nature. Recently, they have demonstrated antifungal, antibacterial, sedative, anxiolytic, antidepressant, antioxidant, and analgesic activities [1,2,3,4,5,6]; however, there are still multiple aspects of essential oils that have not been explored and need unravelling. Inhalation administration is a safe and recognized way of administering essential oils, and it has proven to be effective, especially when addressing the central nervous system (CNS) [7,8,9].

*Chromolaena odorata* (L.) R.M.King & H.Rob (Compositae), previously known as *Eupatorium odoratum* L., is a scrambling perennial shrub native to the Americas, with straight stems which bear three-veined, opposite, ovate-triangular leaves and with a shallow, fibrous root system [10]. It grows up to 2–3 m in height, and can reach up to 5–10 m. Within its native range, *C. odorata* shows marked morphological variability in terms of flower color, leaf shape, and smell of the crushed leaves. In some parts of the world, several forms and their intermediates co-occur, while in others, the population appears homogeneous [11]. *C. odorata* has been the subject of numerous ethnobotanical and ethnopharmacological investigations in the world for its various pharmacological properties such as wound healing activity, antioxidant, stomachic, hemostatic, anti-malarial and antimicrobial activities [12,13,14,15].

The essential oil from the leaves of *C. odorata* (COEO) has frequently revealed a diversity of chemotypes. For example, in a report from Cameroon and Congo [16], *α*-pinene and *p*-cymene were the main compounds whereas, in a sample from India [17], geijerene, *α*-copaene, 3-carene and *β*-caryophyllene have been identified as the major constituents. Another report from Ivory Coast [18] identified pregeijerene and germacrene-D as major compounds. Therefore, the chemical composition of COEO is quite diverse and interesting, which demands further exploration.

In the Republic of Benin (West Africa), which is rich in multiple plant species [19], *C. odorata* is praised for its numerous therapeutic effects, such as its antiseptic and antibacterial activities. The leaves are applied on wounds, which accelerate their healing, and are also used to treat skin irritations. In addition, the inhaled leaves are used to treat respiratory diseases. The plant exhibits a strong fragrance and existing data on its chemical composition suggests the presence of some compounds reported to have sedative activity upon inhalation, such as *α*-pinene or *p*-cymene [3,20]. From the above, we hypothesized that COEO could possess a sedative activity when inhaled. To the best of our knowledge, no study has evaluated the sedative activity of COEO, nor investigated its molecular mechanisms. Therefore, the present study was undertaken to shed light on the sedative activity of COEO. Together with the evaluation of the action mechanisms via the gamma-aminobutyric acid (GABA_A_)-benzodiazepine receptor system, it also investigated the chemical composition of the oil in order to identify the key components responsible for activity. The data reported in the present study adds a plus to the scientific knowledge of the plant and introduces a novel literature for the treatment of CNS and stress-related disorders by using essential oils from plant material. 

## 2. Results and Discussion

### 2.1. Chemical Composition of COEO

The results of the chemical analyses of COEO performed by gas chromatography (GC) and gas chromatography-mass spectrometry (GC-MS) are presented in Table 1. In total, about 15 compounds were identified, representing more than 95% of the oil. A comparison of the composition of COEO in the present study (Republic of Benin, West Africa) with COEOs from different regions of the world was performed, to better understand the various chemotypes existing in *C. odorata*. 

**Figure 1:**
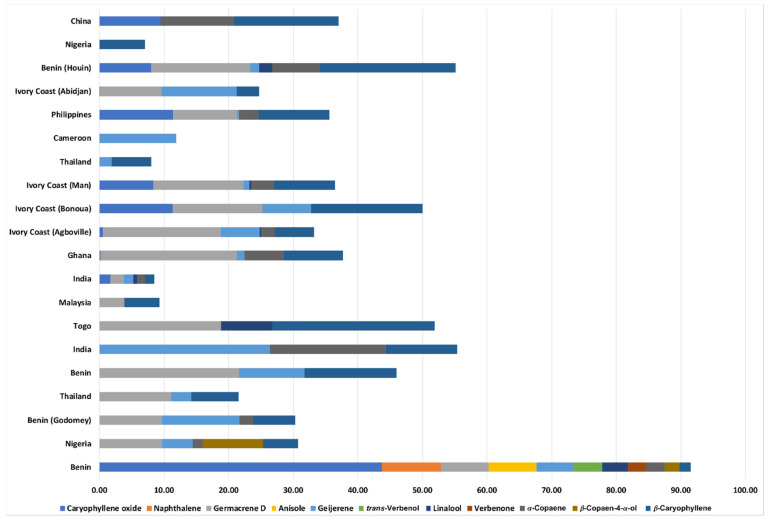
Chemical composition of the main compounds in COEO from our study compared to COEO from other studies in the world. Only components reported in this study are presented in comparison of their ratio with COEOs from other regions in the world. COEO, essential oil obtained from *C. odorata* leaves.

As demonstrated in Figure 1, we found and report here the existence of various chemotypes in COEOs. A study conducted in Nigeria demonstrated that the oil was dominated by *α*-pinene (42.2%), *β*-pinene (10.6%) and germacrene D (9.7%). Another work performed in the Republic of Benin (Godomey) revealed similar composition in addition to pregeijerene (14.6%) as major constituent. Other chemotypes of COEO were represented by geijerene (26.34%), *α*-copaene (17.87%) and *β*-caryophyllene (11.14%) in a sample from India. Phytol (11%) was a major constituent in a sample from Ivory Coast, whereas it was rarely present in other studies and was not detected in our sample. In Thailand, only pregeijerene (40.6%) and dauca-5,8-diene (16.75%) were mainly represented. Similarly, camphor (15.46%) was detected in a sample from Nigeria, and 9,12,15-octadecatrienoic acid, (*Z*,*Z*,*Z*) (25.38%), *n*-hexadecanoic acid (13.34%) and phytol (11.02%) were reported in a study from Malaysia, which emphasizes our argument that *C. odorata* demonstrates a huge variation in the chemical composition of their essential oils. In total, geijerene, pregeijerene, germacrene D, *α*-pinene, *β*-pinene, *β*-caryophyllene and caryophyllene oxide were the compounds frequently reported in all COEO analyzed. However, our study conducted in Abomey-Calavi (Republic of Benin), revealed caryophyllene oxide as the major constituent (43.75%), followed by naphthalene (9.09%). Reasons explaining the chemical variability in COEOs are probably geographical conditions, such as location of the plant, difference in altitude, soil, time of collection or method of extraction of the oil. It is of note to mention that caryophyllene oxide is reported as the major constituent in other Compositae, such as *Artemisia campestris* L. (8.5–38.8%), and other plant species such as *Melaleuca styphelioides* Sm. (43.78%) or *Ocotea acutifolia* (Nees) Mez (56%) [21,22,23]. 

In addition to the comparison between COEOs from different regions, we analyzed the frequency of occurrence of COEOs components. Results were remarkable and demonstrated that all reported COEOs can be categorized into two equal phenotypes: monoterpene hydrocarbon-oil type (frequency = 44.04%) and sesquiterpene hydrocarbon-oil type (frequency = 41.58%). Monoterpene hydrocarbons are represented primarily by pregeijerene and *α*-pinene, and sesquiterpene hydrocarbons are represented by germacrene D and *β*-caryophyllene. The oxygenated sesquiterpenes-oil type is less occurring (frequency = 6.90%) and the key compound in this class is caryophyllene oxide. This strengthens our argument of the huge diversity in COEOs and explains the importance of investigating the chemical composition of essential oils prior to their pharmacological evaluation.

### 2.2. Sedative Activity and Bioactivity-Guided Fractionation of COEO 

**Figure 2:**
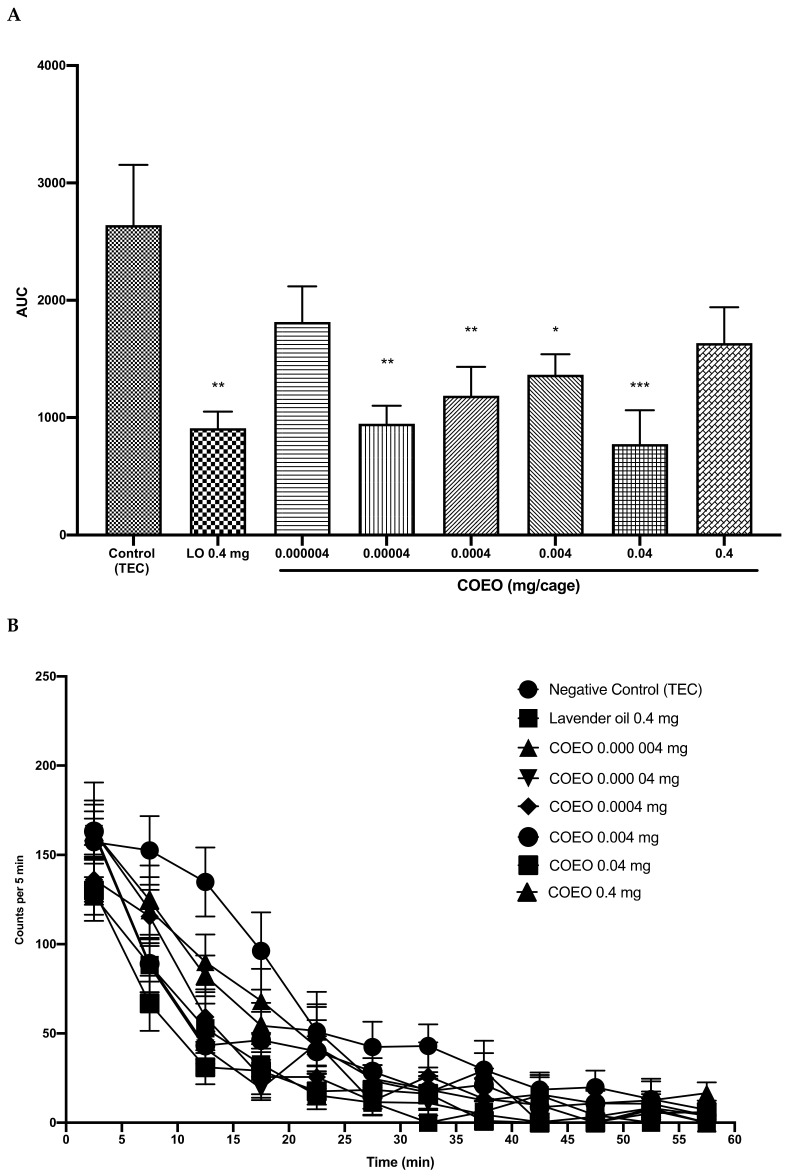
Total spontaneous locomotor activity (**A**) and locomotor activity transition (**B**) of mice treated with COEO (0.000004, 0.00004, 0.0004, 0.004, 0.04, or 0.4 mg/cage). Data are shown as the mean ± SEM of six mice. Statistical differences vs. the control group were calculated using Student’s *t*-test or ANOVA, followed by Dunnett’s test. * *p* < 0.05; ** *p* < 0.01; *** *p* < 0.001. COEO, essential oil obtained from *C. odorata* leaves; TEC, triethyl citrate; LO, lavender oil; AUC, area under the curve; SEM, standard error of the mean; ANOVA, analysis of variance.

In regard to our previous experiments [24,25,26], mice were administered different inhalation concentrations of COEO (0.000004, 0.00004, 0.0004, 0.004, 0.04, or 0.4 mg/cage; volume of the cage 61.2 L). Lavender oil was used as positive control and triethyl citrate (TEC) as a negative control. As shown in Figure 2A, lavender oil significantly decreased locomotor activity by more than 66% (*p* = 0.001) in comparison to the control group. COEO decreased locomotor activity at concentrations from 0.00004 to 0.04 mg/cage (F [7,40] = 4.497; *p* < 0.001), describing a double U-shaped curve. The most effective concentrations were 0.04 and 0.00004 mg/cage, which decreased locomotor activity by 71% and 64%, respectively, in comparison to the control group. In all groups, mice started to sleep from about 25 min after inhalation sample administration, until their activity was reduced to zero (Figure 2B). 

**Figure 3:**
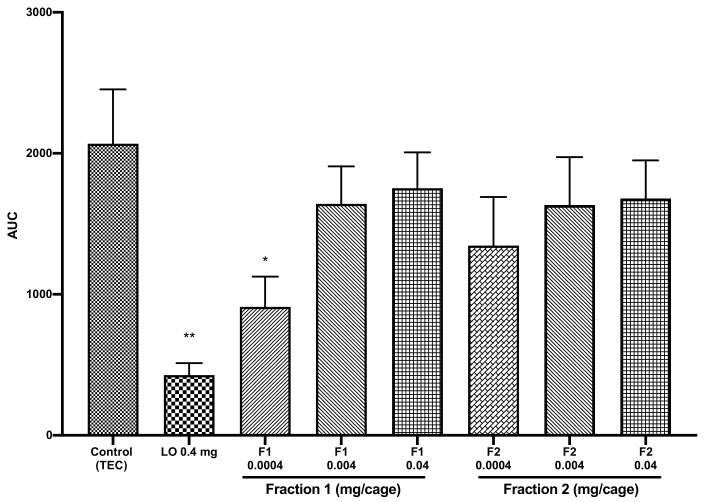
Total spontaneous locomotor activity of mice treated with fractions 1 and 2 (0.0004, 0.004, or 0.04 mg/cage). Data are shown as the mean ± SEM of six mice. Statistical differences vs. the control group were calculated using Student’s *t*-test or ANOVA, followed by Dunnett’s test. * *p* < 0.05; ** *p* < 0.01. TEC, triethyl citrate; LO, lavender oil; AUC, area under the curve; SEM, standard error of the mean; ANOVA, analysis of variance.

To identify the compounds responsible for the decrease in locomotor activity, COEO was fractionated, to give two fractions. Each fraction was analyzed and fraction 1 only induced sedative activity in mice. As shown in Figure 3, it significantly decreased locomotor activity at 0.0004 mg/cage by 56% (*p* = 0.03), compared to the control group. Because fraction 1 had caryophyllene oxide as the major compound, we hypothesized that caryophyllene oxide could be the compound causing the COEO sedative activity.

### 2.3. Caryophyllene Oxide Induced Sedative Activity in Mice

**Figure 4:**
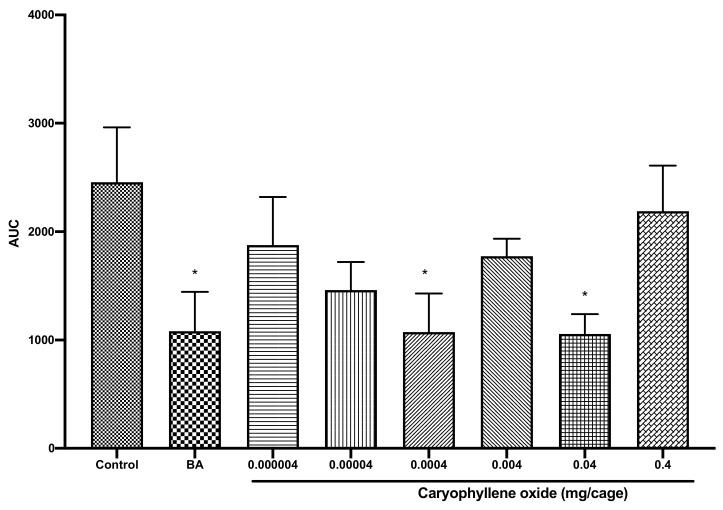
Total spontaneous locomotor activity of mice treated with caryophyllene oxide (0.000004, 0.00004, 0.0004, 0.004, 0.04, or 0.4 mg/cage). Data are shown as the mean ± SEM of six mice. Statistical differences vs. the control group were calculated using Student’s *t*-test or ANOVA, followed by Dunnett’s test. * *p* < 0.05. TEC, triethyl citrate; AUC, area under the curve; SEM, standard error of the mean; ANOVA, analysis of variance.

Caryophyllene oxide was administered to the mice at the same concentrations as COEO. Benzylacetone was used as positive control [24,27], and as shown in Figure 4, it significantly decreased locomotor activity (*p* = 0.04), thus validating the experimental model. In the present study, caryophyllene oxide significantly decreased locomotor activity at 0.0004 and 0.04 mg/cage by 56% and 57%, respectively, compared to the control group (F [7,36] = 2.436; *p* = 0.04) (Figure 4). Likewise, caryophyllene oxide previously induced sedative activity in mice when administered via inhalation at 0.45 mg/cage [28]. In addition, caryophyllene oxide was a major compound of *Baccharis uncinella* DC. essential oil, which demonstrated sedative activity in male albinos mice [29]. In the same way, it also promoted sedation in silver catfish [30,31].

To confirm that caryophyllene oxide truly evaporated in the cage before the mice are inserted, a similar experiment to the open-field arena was performed [24]. The results demonstrated that more than 90% of caryophyllene oxide already evaporated in the air before the mice are inserted, thus confirming that the sedative activity observed is due to the action of caryophyllene oxide. In addition, caryophyllene oxide is the oxidation product of *β*-caryophyllene. Its pharmacological activities include anticarcinogenic, anti-inflammatory, antioxidant, antiviral and analgesic properties [32,33,34]. In previous years, analysis of the essential oil of *Cannabis sativa* L. revealed the presence of mainly sesquiterpene compounds among which caryophyllene oxide was surprisingly identified as a compound that could be sensed by hashish detection dogs [35].

A concentration of 0.04 mg/cage caryophyllene oxide seemed to be more sedative and the results described a double U-shaped dose–response curve. We have previously reported such a dose–response curve [3,4,24], which appears to be the effect of the presence of multiple receptors with different affinities [36]. Comparison of the results of caryophyllene oxide with those of COEO showed that COEO was more sedative at lower concentrations. As a matter of fact, COEO 0.00004 mg/cage was statistically significant, although the described double U curve was similar to that of caryophyllene oxide. Previous studies have agreed that administration of the whole essential oil from plant material is more beneficial than sole administration of their separate compounds [3,24,27]. In the present study, COEO was demonstrated to be more beneficial than the individual administration of caryophyllene oxide. One possible explanation is that in COEO, different compounds are mixed in different ratios, and they can act in synergy to improve the effects of COEO. Accordingly, Fujiwara et al. [37] showed that a mixture of different active oils was beneficial to individual oils, because of a possible synergistic action.

### 2.4. Effect of Caryophyllene Oxide on Pre-Excited Mice with Caffeine

**Figure 5:**
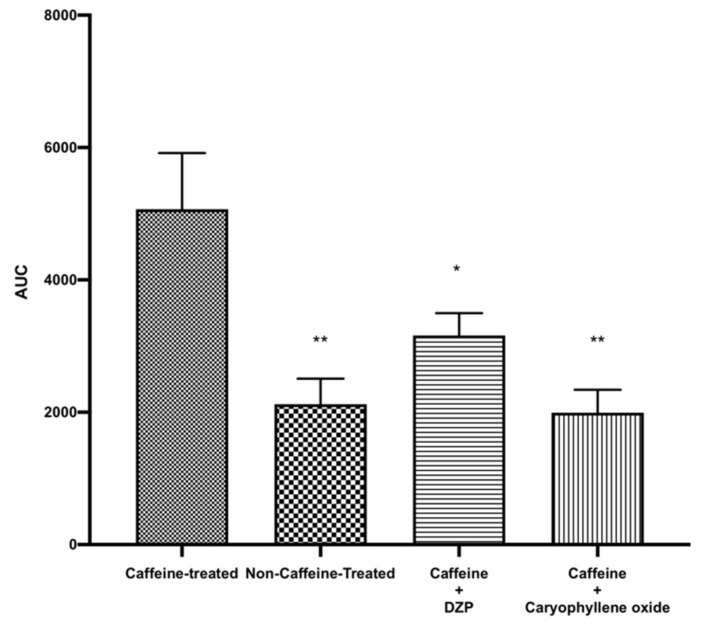
Total spontaneous locomotor activity of mice excited with caffeine (25 mg/kp intraperitoneally) and treated with caryophyllene oxide (0.04 mg/cage). Data are shown as the mean ± SEM of six mice. Statistical differences vs. the control group were calculated using Student’s *t*-test or ANOVA, followed by Dunnett’s test. * *p* < 0.05; ** *p* < 0.01. DZP, diazepam; AUC, area under the curve; SEM, standard error of the mean; ANOVA, analysis of variance.

To better understand the mechanisms underlying the sedative activity of caryophyllene oxide, we first investigated its action on the CNS. Caffeine was administered to mice to induce excitation and, thereafter, caryophyllene oxide (0.04 mg/cage) was given to the mice. As demonstrated in Figure 5, caffeine increased the mice locomotor activity by more than 68% (*p* = 0.003). Similar to diazepam (positive control), upon administration of caryophyllene oxide, mice locomotor activity was decreased by 61% (*p* = 0.001), indicating that caryophyllene oxide is a powerful CNS depressant. In previous studies, monoterpenes and sesquiterpenes were also effective in decreasing the excitation provoked by caffeine [3,38].

### 2.5. Effects of Caryophyllene Oxide on the Pentobarbital-Induced Sleeping Test in Mice

**Figure 6:**
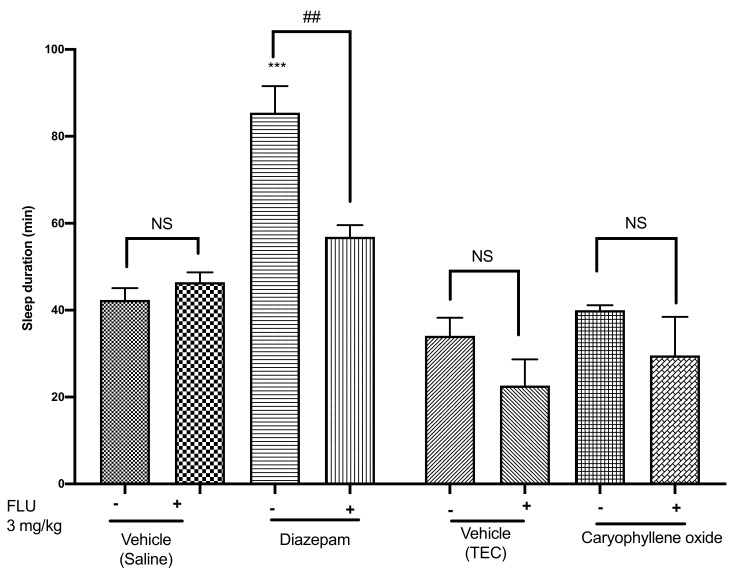
Sleep duration in the pentobarbital-induced sleeping test of mice treated with caryophyllene oxide (0.04 mg/cage), diazepam (3 mg/kg), or flumazenil (3 mg/kg). Data are shown as the mean ± SEM of six mice. Statistical differences vs. the control group were calculated using Student’s *t*-test or ANOVA, followed by Dunnett’s test. *** *p* < 0.001 vs. the control group; ^##^ *p* < 0.01 for flumazenil treatment vs. treatment without flumazenil. TEC, triethyl citrate; FLU, flumazenil; NS, not significant; SEM, standard error of the mean; ANOVA, analysis of variance.

In an attempt to verify the action of caryophyllene oxide on the GABAergic receptor system, mice were administered inhaled caryophyllene oxide after pentobarbital injection. Diazepam, the positive control, prolonged the pentobarbital-induced sleeping time by 102% (*p* < 0.001) and the effect was reversed by 33% following flumazenil administration (Figure 6).

However, caryophyllene oxide only prolonged the sleeping time of mice by 17% and this effect was not statistically significant. Moreover, administration of flumazenil did not change the sleeping time, which suggested that caryophyllene oxide does not operate via the GABAergic receptor system. Conversely, a work in which caryophyllene oxide was extracted from *Psidium guajava* L. hexanolic leaves, demonstrated that it increased the sleeping time induced by pentobarbital [39]. In this study, the authors utilized different dosage and methodology and caryophyllene oxide obtained only in traces was administered intraperitoneally whereas our study was performed on pure caryophyllene oxide administered via inhalation. These differences could explain the divergences in our studies. Although we suggest that the action mechanism of caryophyllene oxide is not facilitated by the GABAergic receptors system, other monoterpene and sesquiterpene compounds such as ascaridole or aristolen-1(10)-en-9-ol have previously demonstrated good sedative activity via the GABA_A_ receptors system [4,40,41]. For instance, numerous studies have suggested the action mechanism of caryophyllene oxide to be mediated via the cannabinoid (CB) receptor system, especially the CB2 receptor [42].

**Figure 7:**
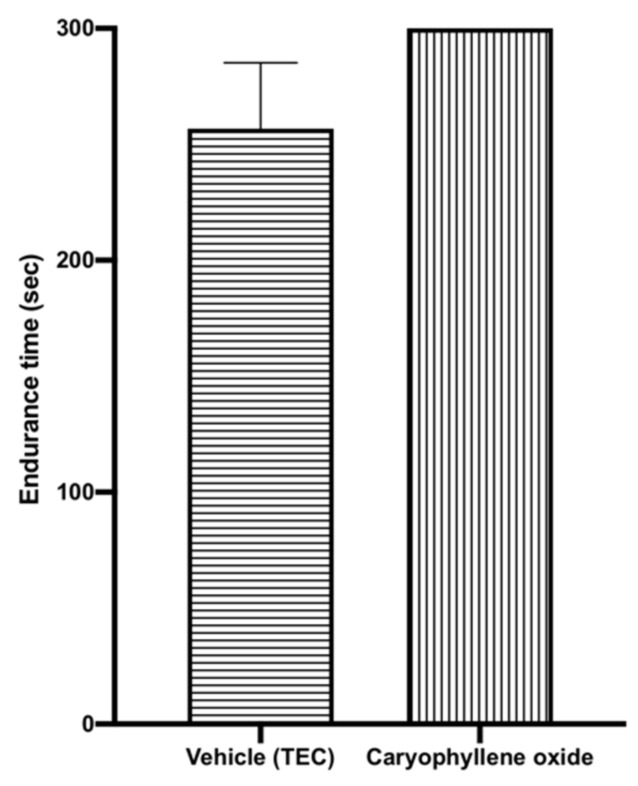
Endurance time in the Rota-rod test of mice treated with caryophyllene oxide (0.04 mg/cage). Data are shown as the mean ± SEM of six mice. Statistical differences vs. the control group were calculated using Student’s *t*-test or ANOVA, followed by Dunnett’s test. TEC, triethyl citrate; SEM, standard error of the mean; ANOVA, analysis of variance.

Further, mice were subjected to the Rota-rod test to confirm that the sedative activity of caryophyllene oxide was not the effect of a deficit in motor function. The results demonstrated that there was no significant change in the latency of mice to fall compared to the control group (*p* = 0.16). Therefore, it follows that the decrease in locomotor activity in our study is not associated with motor incoordination (Figure 7).

### 2.6. Structure–Activity Relationships of Caryophyllene Oxide and Its Precursor Β-Caryophyllene

Caryophyllene oxide is the oxidation product of *β*-caryophyllene, and both compounds are frequently reported in COEOs (frequency of occurrence = 6.15% and 12.01%, respectively). More, *β*-caryophyllene and caryophyllene oxide are natural substances approved as flavoring agents by the Food and Drug Administration (FDA) and by the European Food Safety Authority (EFSA). Therefore, this implied that it could be of interest to compare their physical properties and activities. Analysis of their physicochemical properties (Table 2) [43,44] indicated that both compounds are low volatiles with high affinity to the non-aqueous layer of membranes.

For this reason, when absorbed by the olfactory cells, they can stick to the nasal mucosa and exert their activity. More, they are considered safe with no reported toxicity in studies. As a matter of fact, the median lethal dose (LD_50_) of *β*-caryophyllene and its oxide are both >5000 mg/kg in mice, which is times-fold the highest dose administered in our study, indicating that the sedative activity in our study is exempt of any sign of toxicity.

*β*-caryophyllene is a sesquiterpene compound commonly reported in essential oils from plants such as *Cinnamomum verum* J.Presl and *Syzygium aromaticum* (L.) Merr. & L.M. Perry [45]. In plants, *β*-caryophyllene is sometimes found with isocaryophyllene or its oxidation product, caryophyllene oxide. Studies have elucidated its action mechanism as a selective agonist of the CB2 cannabinoid receptors with no interaction with the CB1 receptors. More, it is considered as a dietary cannabinoid of pharmaceutical promise with multiple pharmacological activities [46]. Its biological effects include anti-inflammatory, anticarcinogenic, antimicrobial, antioxidant, and analgesic activities [47,48,49].

**Figure 8:**
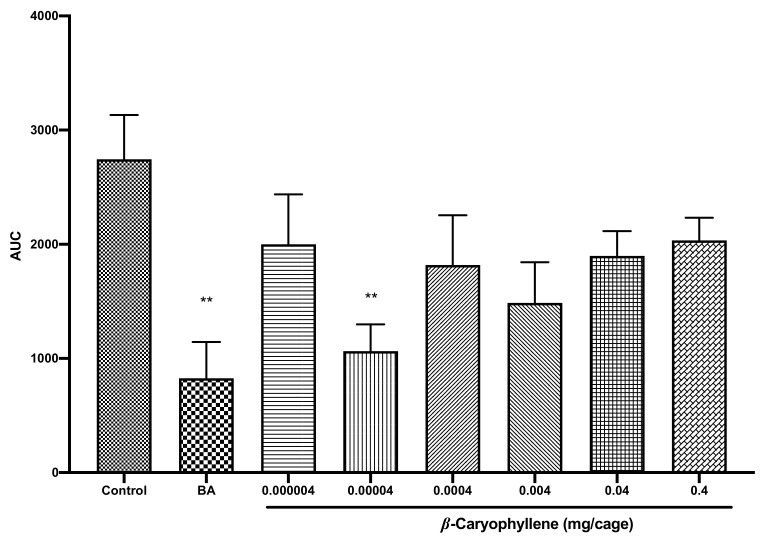
Total spontaneous locomotor activity of mice treated with *β*-caryophyllene (0.000004, 0.00004, 0.0004, 0.004, 0.04, or 0.4 mg/cage). Data are shown as the mean ± SEM of six mice. Statistical differences vs. the control group were calculated using Student’s *t*-test or ANOVA, followed by Dunnett’s test. ** *p* < 0.01. TEC, triethyl citrate; AUC, area under the curve; SEM, standard error of the mean; ANOVA, analysis of variance.

We investigated herein the sedative activity of *β*-caryophyllene. In our open field test, *β*-caryophyllene, in general, demonstrated a reduction in locomotor activity of mice. However, these effects were only statistically significant at a lower concentration of 0.00004 mg/cage, which is lower than that of caryophyllene oxide. As presented in Figure 8, mice locomotor activity was reduced by more than 61% in regard to the control group (*p* = 0.009).

**Figure 9:**
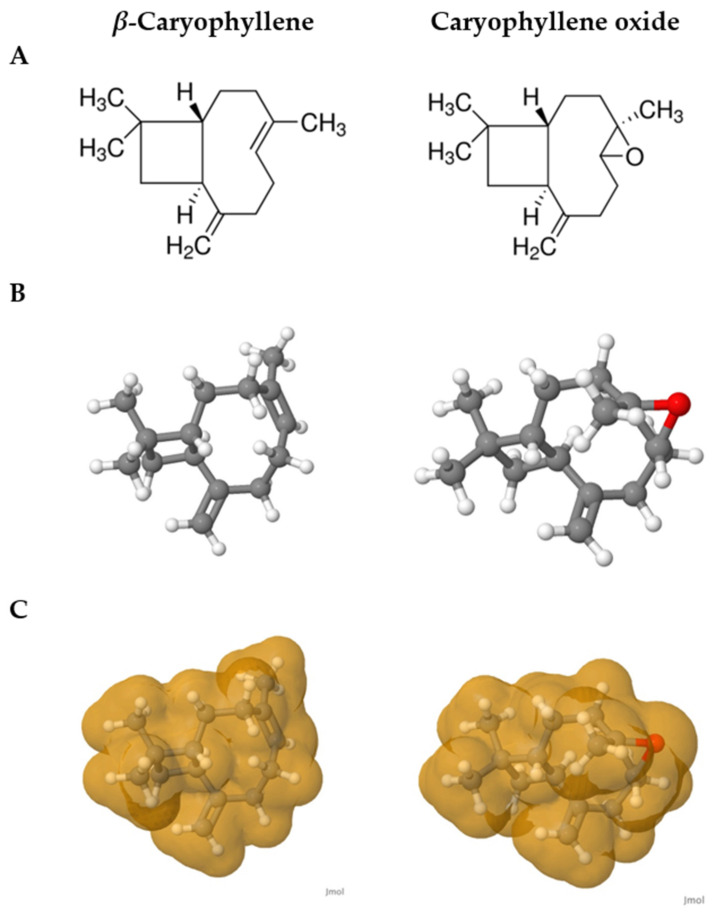
Comparison of the structures of *β*-caryophyllene and caryophyllene oxide. Bidimensional structures (**A**); tridimensional structures (**B**); tridimensional structures with Van der Waals forces (**C**). The sole difference between the structures is the epoxy group in caryophyllene oxide. Structures were drawn by using Jmol [52].

In the same way, *β*-caryophyllene was also reported sedative in a previous study [28] and more, it demonstrated sedative and anxiolytic activities following oral administration to mice [50]. Comparison with the results of caryophyllene oxide revealed the same double U-shaped curve pattern; however, as *β*-caryophyllene seems to be more effective at lower doses, this indicated that the existence of an epoxy functional group (Figure 9A) could render the activity less effective in caryophyllene oxide. Ogawa et al. [28] also previously made similar assertions in their study on *Zingiber zerumbet* (L.) Roscoe ex Sm. related compounds. Moreover, the tridimensional structure of both compounds (Figure 9B,C) indicated that the nine-membered ring cycle in caryophyllene oxide makes it a more complex structure, which does not facilitate penetration to the biological layers. *β*-Caryophyllene on the other side, has a C=C liaison in the nine-membered ring, and is freer to bind to receptors, leading to better reactivity. Recent papers have indicated that the action mechanism of *β*-caryophyllene does not involve GABA_A_/benzodiazepine nor 5-HT 1A receptors, but could be mediated via the cannabinoid CB2 receptors system, as it was also suggested for caryophyllene oxide [50,51]. Further, new studies suggest that CB2 receptors could be involved in the treatment of emotional behaviors such as anxiety [51], which could explain the sedative potential of *β*-caryophyllene and caryophyllene oxide. Overall, *β*-caryophyllene is quite similar to its oxide, and more investigations on these particular sesquiterpenes could broaden actual knowledge on their cellular targets.

## 3. Materials and Methods

### 3.1. Plant Material

Fresh leaves of *C. odorata* were collected in October 2016 from Abomey-Calavi in the Republic of Benin (West Africa) and air-dried. Identification was confirmed by Gaudence Julien Djego of the Laboratory of Botany and Applied Ecology, University of Abomey-Calavi (Benin), and vouchers were deposited in the Herbarium of Experimental Station for Medicinal Plants, Graduate School of Pharmaceutical Sciences, Kyoto University, Japan (specimen number EST-5038), and the National Herbarium of Benin (specimen number AA 6675/HNB).

### 3.2. Preparation and Fractionation of COEO

We extracted COEO by hydrodistillation of 66.98 g of *C. odorata* leaves for 3 h using a Clevenger apparatus, as recommended in the Japanese Pharmacopoeia (JP17; http://jpdb.nihs.go.jp/jp17e/; accessed on 17 August 2020). COEO (690 mg; yield 1.03%) was captured in hexane, dried over anhydrous sodium sulfate, and then concentrated. The headspace of the oil was analyzed by solid phase microextraction (SPME)-GC-MS to confirm that it was hexane free. The obtained oil was stored in sealed vials at 4 °C until analysis.

For fractionation, COEO was subjected to silica gel column chromatography (column diameter 16 mm; column height 170 mm), and the column was eluted with about 250 mL of hexane/acetone (4:1) to obtain fractions 1 and 2. Before the behavioral investigations, each fraction was evaporated until there was no solvent remaining.

### 3.3. Drugs and Reagents

Lavender oil, benzylacetone, and diazepam, all of >95% purity, were obtained from Tokyo Kasei (Japan) and used as positive controls. TEC (Merck, Darmstadt, Germany), a non-sedating odorless solvent, was used to dissolve fragrant components. Caryophyllene oxide (purity ≥ 95%) was purchased from Wako Pure Chemical Industries (Osaka, Japan) and *β*-caryophyllene (purity > 90%) was purchased from Tokyo Chemical Industry Co. LTD (Tokyo, Japan). All chemicals used were of the highest grade available.

### 3.4. Animals

Four-week-old male ddY mice (body weight 20–30 g) were purchased from Japan SLC (Shizuoka, Japan). The mice were housed in colony cages under a 12/12 h light/dark cycle at 25 ± 2 °C and a relative humidity of 50–60%. They were fed pellet chow and given water ad libitum and allowed to acclimatize to these conditions for 1 week before experiments. Animal experiments were conducted in compliance with the guidelines of the Animal Research Committee of Kyoto University (Kyoto, Japan; approval no. 2014-14-3). Experimental procedures involving animals and their care were conducted in compliance with institutional guidelines and the Fundamental Guidelines for Proper Conduct of Animal Experiment and Related Activities in Academic Research Institutions under the jurisdiction of the Ministry of Education, Culture, Sports, Science and Technology, Japan (2006). All experiments were conducted between 10:00 a.m. and 5:00 p.m. under identical conditions.

### 3.5. GC and GC-MS Analyses

We performed qualitative analysis of COEO using a Shimadzu GCMS-QP2020NX gas chromatograph mass spectrometer connected to a Nexis GC-2030 system (Shimadzu Corporation, Kyoto, Japan). We used the following operating conditions: fused silica capillary column, DB-wax (HP), 60 m × 0.25 mm × 0.25 μm; column temperature starting at 60 °C and increasing at a rate of 3 °C/min until 220 °C; injector temperature, 210 °C; carrier gas, helium, 25 cm/s; column head pressure, 100 kPa; ionization energy, 70 eV; injection volume, 1.0 μL; MS interface temperature, 150 °C/min; MS mode, electron impact (EI); detector voltage, 0.4 kV; mass range, 35–300 u; and scan speed, 300 u/s.

Quantitative analysis was performed using a Hitachi G-5000 GC system (Hitachi, Ltd., Ibaraki, Japan) equipped with a flame ionization detector (FID) as follows: fused silica capillary column, TC-wax (HP), 60 m × 0.25 mm × 0.25 μm; column temperature, same as for GC-MS; injector temperature, 200 °C; detector, FID, 220 °C; carrier gas, helium, 0.8 mL/min; split ratio, 100:1; column head pressure, 200 kPa; and injection volume, 1 μL. We determined the linear retention indices of each constituent using *n*-alkanes as standards and identified chemical compounds by comparing their retention indices (RIs) and mass spectra (MS) using NIST Special Database 2 (https://www.nist.gov/srd/nist-special-database-2; accessed on 19 October 2020) or by comparison with authentic samples.

### 3.6. Open-Field Test

The sedative effects of COEO were evaluated in mice (*n* = 6/group) using an open-field test, as previously described [4,24,25]. Briefly, we fixed four pieces of filter paper in the four corners inside a glass cage (60 cm wide, 30 cm long, 34 cm high) using adhesive tape. We deposited a sample on each piece of filter paper and closed the cage so that the vapor pervaded by natural diffusion. After 1 h of charging the samples, we placed a mouse in the center of the cage and monitored it by video for another 1 h. During monitoring, we counted the frequency each mouse crossed lines drawn at 10 cm intervals on the cage floor every 5 min. In addition, we calculated the AUC of locomotor activity counts per 5 min (*y*-axis) and time (*x*-axis) representing the total locomotor activity by the trapezoidal rule.

### 3.7. Caffeine-Induced Stimulation of Locomotor Activity in Mice

We intraperitoneally administered caffeine to mice (*n* = 6/group) at a dose of 25 mg/kg. Next, we placed each mouse in a cage filled with vapor from the sample solution 30 min after administration and measured locomotor activity for another 1 h, as described in the open-field test. Diazepam (5 mg/kg) used as a positive control was administered orally 30 min before caffeine administration. The test was performed as described by Takemoto et al. [41] with modifications.

### 3.8. Pentobarbital-Induced Sleeping Test

We administered sodium pentobarbital (42 mg/kg) intraperitoneally to mice (*n* = 6/group) and immediately placed them in a cage filled with vapor of the sample solution (400 μL/cage). Sleeping time was defined as the time difference between loss and recovery of the righting reflex. Diazepam (3 mg/kg) used as a positive control was administered orally 30 min before sodium pentobarbital injection [41]. To evaluate the action mechanism of molecules in the CNS, 3 mg/kg of flumazenil, a specific GABA_A_-benzodiazepine receptor antagonist, was administered intraperitoneally 15 min before diazepam administration.

### 3.9. Rota-Rod Test

A Rota-rod treadmill (MK-600; Muromachi Kikai Co., Ltd., Japan) was used to perform the experiment following the methodology of Takemoto et al. slightly modified [41]. To adapt the mice to the Rota-rod, the mice (*n* = 6/group) underwent a 2-day training period during which they were placed on the rotating rod first at 5 rpm for 3 min (mode 1) and then at 40 rpm for a total of 3 trials/day. Mice that failed to remain on the rotating rod after two trials were excluded. On day 3, the Rota-rod test was performed. After 60 min of sample inhalation, the mice were placed on the rotating rod, and the duration (up to 3 min) that the mice remained on the rotating rod was recorded.

### 3.10. Statistical Analysis

Statistical analyses were performed using Student’s *t*-test or one-way analysis of variance (ANOVA), followed by Dunnett’s multiple-comparison test using GraphPad InStat version 8.0 (GraphPad Software, San Diego, CA, USA). All values were expressed as the mean ± standard error of the mean (SEM). *p* < 0.05 was considered statistically significant.

## 4. Conclusions

In the present study, we have demonstrated an important variation in the essential oil composition of COEOs from different regions in the world. COEO from the Republic of Benin was investigated for sedative activity and its major constituent, sesquiterpene caryophyllene oxide, revealed to be a key component in the sedative activity. In an attempt to shed light on the mechanisms of action, we demonstrated that the GABAergic receptors system was not involved in the sedative activity, and suggested that binding to other systems, such as the cannabinoid receptor system, could explain the activity. Therefore, further investigations on the action mechanism of caryophyllene oxide and similar compounds is needed.

In total, COEO and its sesquiterpene components could be potentially used in the treatment of CNS-related disorders, and in a forthcoming article, we will investigate the structure-activity relationship of selected sesquiterpenes compounds, to better understand their pharmacological activities and action mechanisms in the CNS.

## Figures and Tables

**Table 1:** Chemical composition of COEO.

N°	Compounds ^a^	Peak Area (%) ^b^	Calculated RI ^c^	Literature RI ^d^
1	Geijerene	5.68	1298	1338
2	*α*-Copaene	2.84	1461	1491
3	Benzene, 1,2,4-triethyl-	1.14	1501	1501
4	Linalool	3.98	1504	1543
5	Pinocarvone	1.70	1535	1575
6	*β*-Caryophyllene	1.70	1559	1588
7	*β*-Copaen-4-*α*--ol	2.27	1571	1594
8	*trans*-Pinocarveol	0.57	1611	1661
9	Anisole	7.39	1627	
10	*trans*-Verbenol	4.55	1633	1680
11	Germacrene D	7.39	1661	1708
12	Verbenone	2.84	1664	1720
13	*p*-Mentha-1,5-dien-8-ol	0.57	1673	1738
14	Naphthalene	9.09	1690	1755
15	Caryophyllene oxide	43.75	1916	1986
	Unidentified	4.55		
	Total	100		

^a^ Order of elution determined using a DB-wax column. ^b^ Peak area percentage determined by calculating the peak area of the FID chromatogram in GC analyses. ^c^ Retention index, calculated against C_10_–C_26_ *n*-alkanes on a DB-wax column. ^d^ Retention index, taken from the NIST library. COEO, essential oil obtained from *Chromolaena odorata* leaves; RI, retention index; GC, gas chromatography.

**Table 2:** Physicochemical properties of *β*-caryophyllene and caryophyllene oxide.

	*β*-Caryophyllene	Caryophyllene Oxide
Appearance	Colorless to pale yellow clear oily liquid	Pale yellow white crystalline solid
Molecular Weight	204.35 g/mol	220.35 g/mol
Formula	C_15_ H_24_	C_15_ H_24_ O
Odor	Spicy	Woody
Optical Rotation	−5.00 to −10.00	−65.00 to −75.00
Melting Point	129.00 to 130.00 °C at 14.00 mm Hg	60.00 to 62.00 °C at 760.00 mm Hg
Boiling Point	254.00 to 257.00 °C at 760.00 mm Hg	279.68 °C at 760.00 mm Hg
Vapor Pressure	0.013000 mmHg at 25 °C	0.007000 mmHg at 25 °C
Flash Point	205.00 °F	>212.00 °F
logP (*o*/*w*)	6.777	4.429

## Data Availability

Data sharing not applicable.
